# Putney to Mortlake: Competitive Athletics

**Published:** 1912-03-30

**Authors:** 


### PUTNEY TO MORTLAKE: COMPETITIVE ATHLETICS.

Every now and then the sport-loving public has its attention aroused by the early, and sometimes the sudden death of some well-known oarsman, footballer, or track athlete. Not unnaturally the question is thereupon raised whether the cult of athletics may be responsible for some of these premature terminations of lives that ought, one might think, to be exceptionally robust and long-lived. The problem is a very intricate one, for it is complicated by so many factors, the effect of which does not appear on the surface. Each case requires the most careful consideration on its merits, in order that the exact importance may be attached to each of the various causes which may have influenced the course of each individual case. Statistics, therefore, are more than usually capable of fallacy in any endeavour to estimate the longevity or otherwise of those whose devotion to, and success in, athletic exercises has been strongly marked.

While this has to be borne steadfastly in mind, the statistical tables recently published in the United States concerning the naval officers who passed through the Naval Academy into the Navy of the Republic during the years 1891 to 1911, have probably a greater value than most similar unsifted figures could possess. For it may be assumed that practically the whole of these cadets were, at the commencement of their course, sound in wind and limb; this would be ensured by the medical examination at entry. Then also the subsequent careers of the cadets must have run along fairly similar lines, for life in the Navy is much the same for all. It is true that the intervention of the United States in Cuba, Porto Rico, the Philippines, and other tropical countries has introduced a climatic influence into the life histories of naval officers; and that malaria, yellow fever, and other tropical disorders must have disturbed the vital statistics to a certain extent. And it is also true that syphilis, over-indulgence in tobacco or alcohol, zymotic diseases, exposure to the weather, and similar factors have to be taken into account just as much afloat as ashore. But in the main the life is a healthy one, especially for officers, and at least the incidence of the factors mentioned must be fairly equal through the Service. The figures themselves, then, may be expected to be instructive; or, at any rate, less fallacious than most similar compilations could be. The Surgeon-General of the Navy has examined the after-history of six hundred and twenty-five officers whose early career was signalised by exceptional athletic proficiency and distinction: inasmuch as the oldest of these passed out of the Academy in 1891, it is fair to regard them as a group of men still in the prime of life. Twenty-one are dead; and two of these fatalities were due to cardiac disease believed to have been engendered by athletics. Of the other 604 it is stated that almost a third have been at one time or another on the sick list for reasons possibly or probably due to the same cause. Forty-eight of them have been noted as the subjects of actual valve-lesion, murmurs, irregular cardiac action, dilatation, or arterial disease. Fifteen suffer or have suffered from hernia; and this condition is said to be chiefly noticeable among those who devoted themselves particularly to football. Tuberculosis accounts for seventeen more of the casualty list.

The Bureau of Medicine and Surgery has come to the conclusion that it is not so much the games and competitions themselves that do the harm, as the over-training and too prolonged periods of preparation that the crack athletes indulge in. Long-distance racing is held to be the most injurious of all sports; and Marathon racing is regarded as the worst of all. With these conclusions it is easy to sympathise; they contain a lesson which this country may well take to heart. Yet it is doubtful whether the mischief thus done is anything like so great in this country as in the States. It is an old observation that the American takes his sports much more seriously than we do, and thereby can often defeat his English competitor. To win is the great ambition over there, whereas to have a good game is the chief motive here. In Great Britain probably the Oxford and Cambridge Boat-race is the event which demands of its protagonists the most prolonged and arduous training; yet famous Blues have a knack of living to be octogenarians, or even longer still. It is true that about twelve years ago three still young Oxford Blues died within three or four years; but, broadly speaking, rowing Blues are notorious for longevity. This may be due to the great care with which a medical examination of every man is conducted before he is allowed to start training. It is probable, therefore, that the evil pointed out in the Surgeon-General's report is one much less prevalent in this country, though it does exist in some degree.

